# Augmented feedback for powered wheelchair training in a virtual environment

**DOI:** 10.1186/s12984-019-0482-3

**Published:** 2019-01-18

**Authors:** Catherine Bigras, Dahlia Kairy, Philippe S. Archambault

**Affiliations:** 1 Integrated Program in Neuroscience, McGill University, Montreal, Canada; 2 Interdisciplinary Research Center in Rehabilitation (CRIR), Montreal, Canada; 3 École de réadaptation, Faculté de Médecine, Université de Montréal, Montreal, Canada; 4 School of Physical and Occupational Therapy, McGill University, Montreal, Canada

**Keywords:** Augmented feedback, Powered wheelchair, Training, Virtual reality

## Abstract

**Background:**

Powered wheelchair (PW) driving is a complex activity and requires the acquisition of several skills. Given the risks involved with PW use, safe and effective training methods are needed. Virtual reality training allows users to practice difficult tasks in a safe environment. An additional benefit is that augmented feedback can be provided to optimize learning. The purpose of this study was to investigate whether providing augmented feedback during powered wheelchair simulator training results in superior performance, and whether skills learned in a virtual environment transfer to real PW driving.

**Methods:**

Forty healthy young adults were randomly allocated to two groups: one received augmented feedback during simulator training while the control group received no augmented feedback. PW driving performance was assessed at baseline in both the real and virtual environment (RE and VE), after training in VE and two days later in VE and RE (retention and transfer tests).

**Results:**

Both groups showed significantly better task completion time and number of collisions in the VE after training and these results were maintained two days later. The transfer test indicated better performance in the RE compared to baseline for both groups. Because time and collisions interact, a post-hoc 2D Kolmogonov-Smirnov test was used to investigate the differences in the speed-accuracy distributions for each group; a significant difference was found for the group receiving augmented feedback, before and after training, whereas the difference was not significant for the control group. There were no differences at the retention test, suggesting that augmented feedback was most effective during and immediately after training.

**Conclusions:**

PW simulator training is effective in improving task completion time and number of collisions. A small effect of augmented feedback was seen when looking at differences in the speed-accuracy distributions, highlighting the importance of accounting for the speed-accuracy tradeoff for PW driving.

## Background

Around 1 % of community-dwelling Canadians use a mobility device (manual wheelchair, powered wheelchair or scooter) [1]. This proportion is higher for the older portion of the population, with around 4 % of the population aged 75 and over using a wheelchair [1]. In comparison to a manual wheelchair, powered wheelchairs (PWs) require almost no physical effort and enable people with more severe limitations to maneuver in their home or in a community environment for extended periods of time. However, PW driving is more cognitively demanding and safety is an important concern. Accidents with PWs are frequent and often result in injuries or damage to the device or the environment [2].

PW driving requires the acquisition of several skills and can be challenging for new users. A recent study interviewed PW users to describe the challenges that they experience in maneuvering their PWs during daily activities [3]. Difficulties encountered included basic tasks, such as joystick control and maneuvering backward, but consisted more of complex tasks performed in a community setting. Challenges included maneuvering in constrained areas (e.g. public washrooms, shopping aisles), going through doorways, avoiding fixed (e.g. garbage can) and moving obstacles (e.g. people), and managing uneven or slippery surfaces. Such situations are difficult to reproduce when training new users in a clinical setting. In addition, it can be difficult to allocate sufficient time for wheelchair training in order to allow new users to master all the skills required to safely maneuver a PW.

Given the safety risks involved and the extensive skillset required for driving a PW, effective training protocols for new PW users are needed. Virtual reality offers many advantages, such as portability and safety. Over the last 10 to 20 years, a number of virtual reality PW simulators have been proposed (see [4] for a review) that range from simple flatscreen interfaces to head mounted displays with 3D stereoscopic vision. The McGill Wheelchair Simulator, or miWe, [5] is a non-immersive virtual environment that runs on a regular computer and provides a first person, 3D perspective view, presented on a computer screen located at eye level. The virtual PW functions like a real PW: it is controlled using a regular joystick, and parameters such as speed and acceleration correspond to a real PW. Several virtual scenarios have been designed, based on an analysis of users’ needs [3], such as entering/exiting an elevator, navigating in a crowded shopping mall, street crossing, etc. Participants receive feedback about collisions and the time required to complete an activity. With the simulator, complex tasks that are difficult to replicate in the real environment, such as maneuvering in a crowded hallway, can be practiced safely and in a realistic manner. An additional benefit of using virtual reality to train PW users is that features in the environment can be manipulated in order to maximize learning. One approach involves providing learners with extrinsic information regarding their performance of a task. Such augmented feedback is given in addition to the usual sensory-perceptual information that learners naturally perceive when performing a task. Augmented feedback regarding the pattern or the outcome of a movement can help learners acquire new skills by facilitating error detection [6–9].

Another important aspect of training that is easily manipulated in a virtual environment is variability of practice. Changing the task parameters during training has been shown to yield superior results in terms of learning than fixed practice [10]. Many variations of a task can be created in a simulator to increase the variability of PW training.

The purpose of PW training is to induce long-term learning in PW users. An important distinction is made between motor learning and performance. Motor learning refers to the long-term changes in the execution of a movement, whereas performance reflects the transient effects of practice [11]. Improvement in performance can be seen during practice of a given task over a number of trials, but these changes may not necessarily reflect motor learning, if there is no retention of the skill once practice has ceased. The initial boost in performance may be dependent on the practice conditions such as the large number of trials and the augmented feedback provided during the acquisition phase [12]. To verify that motor learning has taken place, it is necessary to administer a retention test, sufficiently removed in time from the practice session, in which the learner performs the task without any feedback. The change in the execution of the motor task from before training to the retention test reflects motor learning.

An additional aspect to consider when training with a simulator is whether the skills learned in the virtual environment transfer to real PW driving. To test this, real PW driving performance is assessed before and after simulator training with a task similar to the one performed in the virtual environment. The results of this test reflect the transferability of the skills that were learned in the simulator. Some wheelchair simulators have been shown to be effective for wheelchair training [4, 13–15] (for a recent review, see [16]). However, these studies were conducted with small sample sizes and the real PW driving transfer test was administered immediately following the training session.

The present study sought to determine whether, in naïve healthy adults, providing augmented feedback during simulator training results in better learning and retention of PW skills in comparison to a control group receiving no such feedback; and whether simulator training improves PW performance in the real environment.

## Methods

### Participants

All participants provided informed consent, as approved by the ethics committee of the Interdisciplinary Research Centre in Rehabilitation (CRIR, Canada). Forty healthy participants were recruited for this study. The study took place at the Jewish Rehabilitation Hospital over the course of four months. To be included, participants had to be between the ages of 18 and 50 and be right-handed. Left-handed people were excluded because the real PW used for the experiment was right-handed. The age range was defined as 18–50 at the beginning of the study, but for convenience and homogeneity of sample, only young adults were recruited. Participants were excluded if they had any self-reported motor, sensory or cognitive difficulties that could interfere with PW driving, if they had any previous PW driving experience or if they had significant experience maneuvering a manual wheelchair. Participants were randomized into two groups. Participants in the feedback group received augmented feedback during simulator training, while those in the control group did not receive any augmented feedback during training.

### Study design

**Fig. 1:**
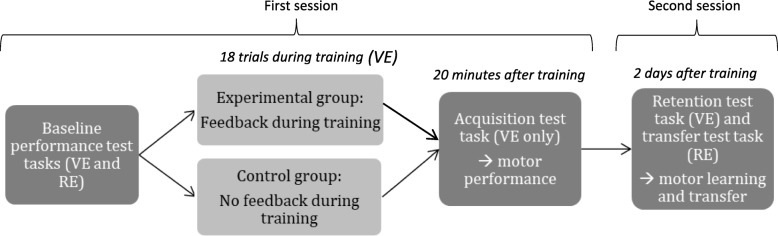
Summary of experimental design

The study design followed a paradigm traditionally used in motor learning studies [11], consisting of an acquisition phase and a retention/transfer phase (Fig. 1). Participants were required to attend two sessions, two days apart. On the first day, participants were asked to complete a demographic questionnaire regarding their age, gender, occupation, and number of years of education. Participants were then introduced to the virtual and real PW. They received basic instructions on how to control the joystick and performed an initial familiarization task in both the virtual (VE) and real environments (RE). Then, their baseline performance was assessed by performing a single trial of a “test” task in each of the VE and RE (described below). Following this initial assessment, participants trained in the VE only. Training for the experimental group consisted of 18 trials with augmented feedback. The control group performed the same amount of training trials, but with no augmented feedback. Twenty minutes after training, participants of both groups were tested again in the VE (single test trial). The purpose of the post-training test was to assess the initial acquisition of motor skills. Two days later, participants returned for a retention test in the VE and a transfer test in the RE, which sought to evaluate whether motor learning had taken place. No feedback was provided during any of the test trials.

### Task design

The task used for training and testing was chosen by taking into account challenges that PW users often encounter, such as maneuvering in confined spaces, avoiding collisions and maneuvering backward [3, 17]. The task was designed to combine these three skills.

### i. VE training task

**Fig. 2:**
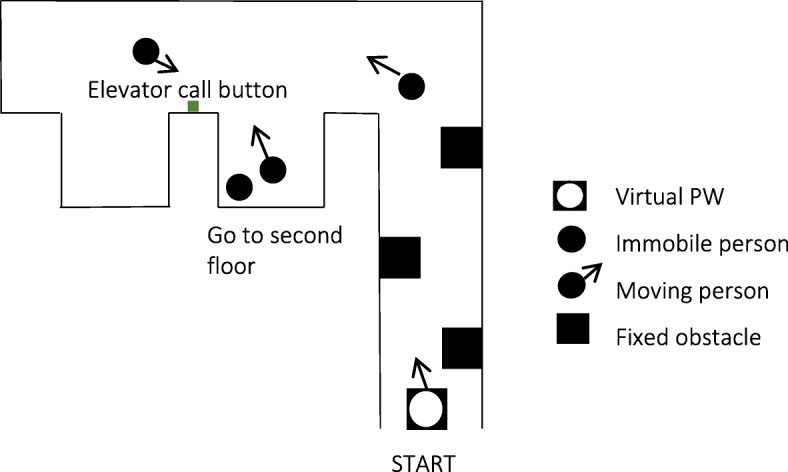
Outline of PW task (VE). Not drawn to scale

The VE training task consisted of navigating through a hallway that contained fixed obstacles, turning around a 90-degree corner, and using an elevator (Fig. 2). Participants had to press on the elevator’s call button, wait for the doors to open, enter the elevator before the doors close, make a U-turn inside the elevator, press on the 2nd floor button and finally exit the elevator once it reached the next floor. Moving obstacles (people) were present in the hallway and the elevator. Participants were instructed to perform the task as quickly as possible while avoiding collisions.

Because variable practice has been shown to yield superior results in terms of learning than fixed practice [10], four versions of the training task of different difficulty levels were created. The training task versions varied in terms of parameters such as hallway width, position of the obstacles, elevator door width, elevator area, and the number of moving virtual characters (Table 1).

**Table 1:** Summary of VE training and test tasks

	VE Task 1	VE Task 2	VE Task 3	VE Task 4	VE Test Task
Hallway	Wide hallway with two fixed obstacles	Regular width hallway with three fixed obstacles	Narrow hallway with two fixed obstacles	Narrow hallway with three fixed obstacles	Regular width hallway with three fixed obstacles
Elevator	Wide elevator	Medium width elevator	Medium width elevator	Narrow elevator	Medium width elevator
Number of moving obstacles	Two	Four	Four	Five	Four

### ii. VE test task

An additional variation of the VE training task was created for the sole purpose of testing performance. This VE test task was a variant of the training tasks and was of intermediate difficulty level. The VE test task was the same for all tests (baseline, acquisition and retention).

### iii. RE test task

**Fig. 3:**
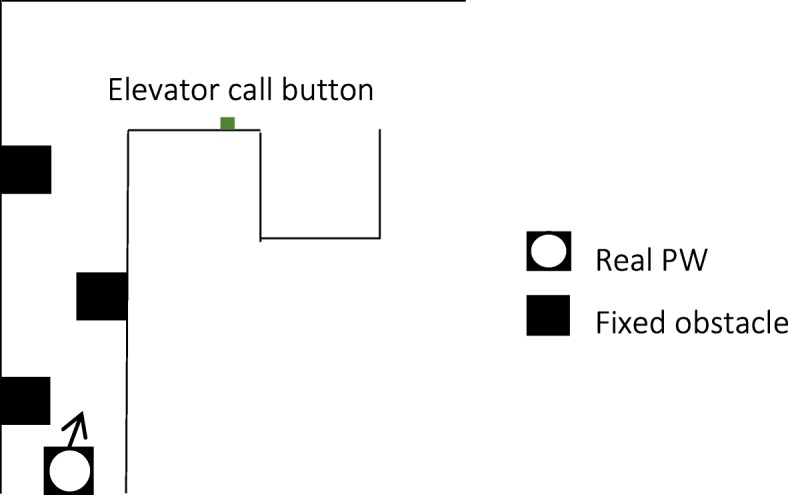
Real PW task outline. Not drawn to scale

The real environment test task was similar to the VE training and test tasks (Fig. 3). Participants drove in a hallway of the hospital while avoiding fixed obstacles (chairs), turned the corner and pressed on the elevator button. As the real PW driving was conducted in a busy hospital, participants were not asked to enter the elevator. For convenience, the task ended when participants pressed the elevator button. There were no moving obstacles for the real PW test task.

Because performance with the real and virtual PW were not directly compared, the RE and VE test tasks did not need to be identical. They had to be similar enough to adequately assess transfer of learning from the VE to the RE.

### Augmented feedback

**Fig. 4:**
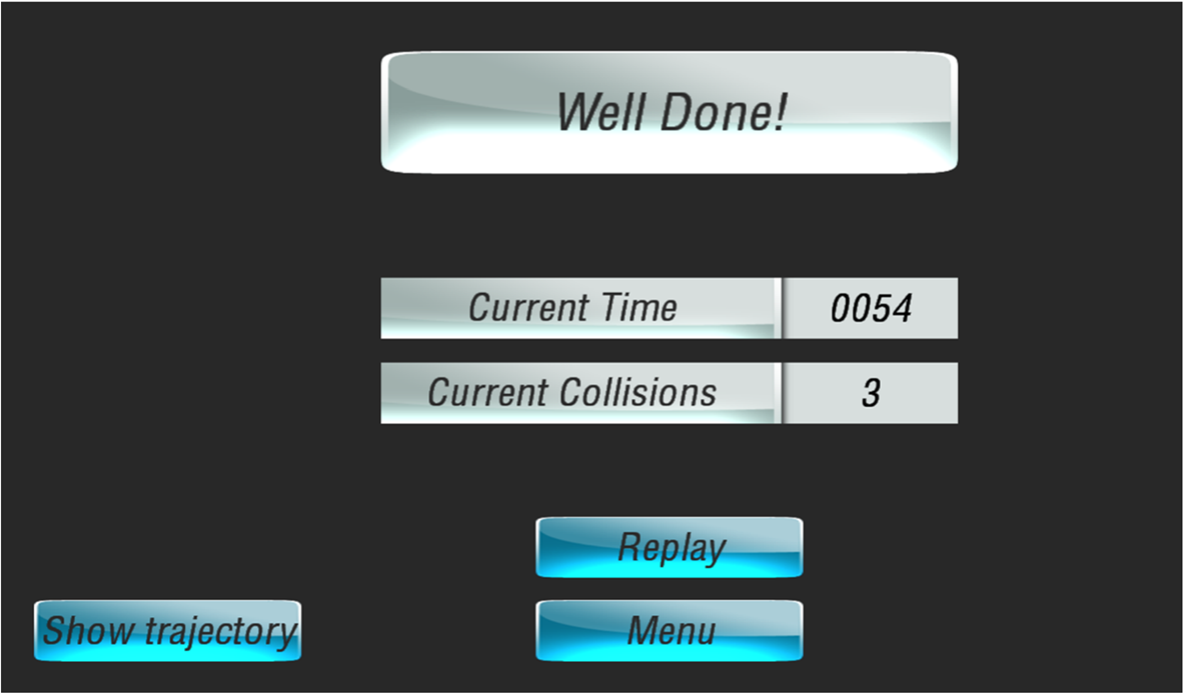
Terminal feedback regarding time and collisions given to feedback group. Additional feedback regarding the pattern of the movement was also provided. The feedback consisted of the participants’ trajectory overlaid on a 2D diagram of the task viewed from above (Fig. 5a). The locations where participants made collisions (red) or came close to making collisions (blue) were indicated on the diagram. The participants were then shown snapshots of their 3 worst moments (see Fig. 5b for example). The images showed what part of the PW made contact or came close to the obstacle

**Fig. 5:**
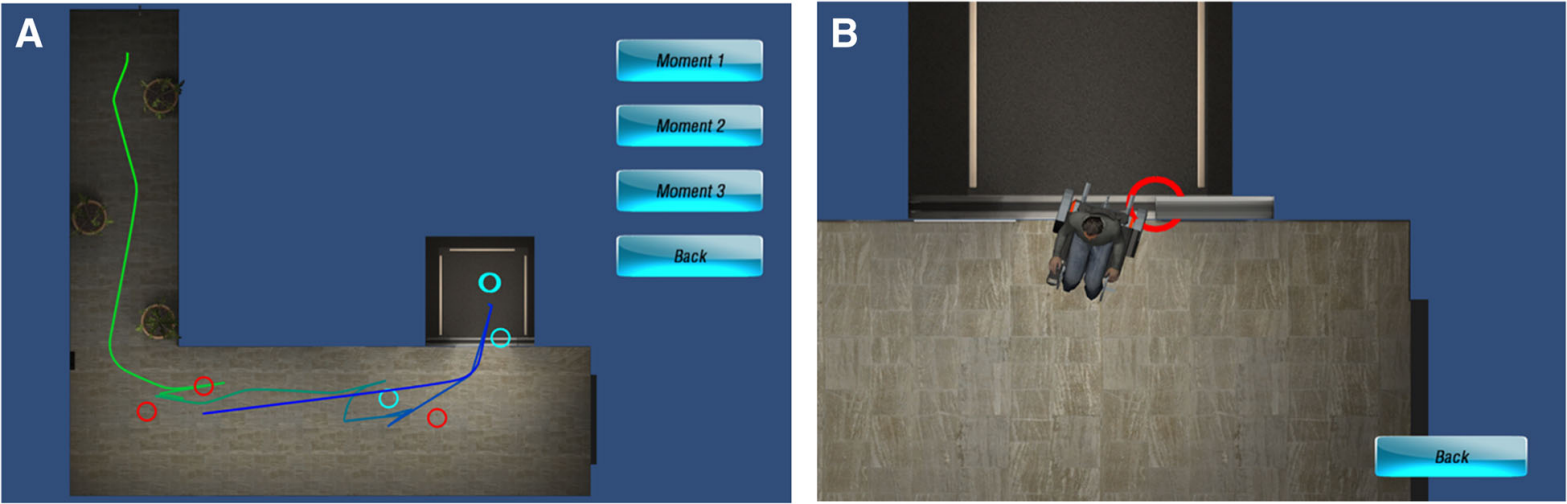
Terminal feedback regarding the PW’s trajectory given to feedback group. Participants can review where in their trial they made collisions (panel **a**, red circles) or came close to making a collision (panel **a**, blue circles); they can then review what part of the wheelchair collided with an obstacle (panel **b**)

Augmented feedback is provided in addition to the normal, intrinsic feedback that is obtained from a task. To provide a realistic learning setting in the simulator, collisions with people and objects were accompanied by different sound effects. Additional, augmented feedback was provided to the feedback group during the VE training tasks only. Augmented feedback is useful when it is informative enough to guide future movement. The appropriate level of information depends on the skill level of the learner and on the task difficulty [18]. PW driving is a complex activity and therefore feedback must be specific enough with regards to the environmental goal to induce changes during training. However, since the participants in this study were novices, we must have ensured not to provide too much information or information that is too complex. Since one of the goals of PW driving is to drive safely (by avoiding collisions) and efficiently (by not driving too slowly), we decided to provide terminal feedback (i.e. at the end of the task) regarding the number of collisions and the time to complete the task (Figs. 4 and 5).

In order not to overwhelm participants with this information, the trajectory information was given on a faded schedule: they received feedback after each of the first three trials and following these, they received it every three trials.

There were four VE training tasks and 18 training trials. The four VE training tasks were ordered in a way to appear random over the course of training so that participants could not guess which task was coming next. Each participant followed the same task order during training. Table 2 summarizes the VE training task versions and feedback schedule for the feedback group for the 18 training trials. The control group followed the same task order but did not receive any feedback.

**Table 2:** Summary of feedback schedule given to feedback group during training

Trial	1	2	3	4	5	6	7	8	9	10	11	12	13	14	15	16	17	18
VE training task	1	2	3	4	2	4	1	3	4	3	2	3	2	1	1	3	2	4
Trajectory feedback (exp. Group only)	x	x	x			x			x			x			x			x
Collision and task completion time feedback (exp. group only)	x	x	x	x	x	x	x	x	x	x	x	x	x	x	x	x	x	x

### Performance outcomes

Participants were instructed to perform the VE training, the VE test task and the RE test task as fast as possible while avoiding collisions. The simulator recorded the task completion time and the number of collisions for each trial. Participants were filmed as they drove in the RE to extract the time for task completion and the number of collisions.

### Data analysis

Means and standard deviations were computed for task completion time and number of collisions (in VE and RE) and distance from the elevator button (RE only) for the baseline, 20-min post-training and retention tests. To analyze differences in task completion time and number of collisions between performance at baseline, acquisition and retention and between the groups, we ran mixed-model analyses of variance using the *mixed* procedure in SPSS 23 (IBM, USA). A mixed effect analysis is a type of linear analysis where the error variance is modeled. It is more flexible than a repeated measures ANOVA and leads to better estimates [19]. We included effects for time, group (feedback/no-feedback) and interaction (time x group). We chose an unstructured variance matrix because it offered the best fit with the model.

## Results

### VE test task

**Fig. 6:**
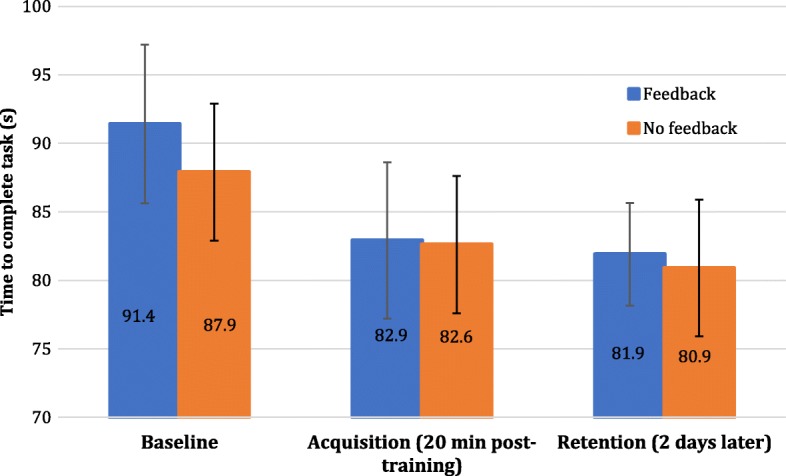
Time to complete task in the VE for feedback and no feedback group at baseline, acquisition and retention. Overall, participants had better performance from baseline to acquisition and the effect was maintained at retention. No significant group differences were found. Error bars represent confidence intervals

The results for the time to complete the VE test task at each time point are shown in Fig. 6. Participants took 90 s on average to complete the VE test task before training. Twenty minutes after training, participants were seven seconds faster than at baseline. The effects seen at acquisition were retained on the retention test two days later, where participants were eight seconds faster than at baseline. The control group had significantly better baseline performance than the feedback group, but their performances on the VE test task were similar at acquisition and retention. The mixed effects model found a significant effect of time from baseline to acquisition (*p* = .002) and from baseline to retention (*p* < .001) for all participants. No significant group differences were found.

**Fig. 7:**
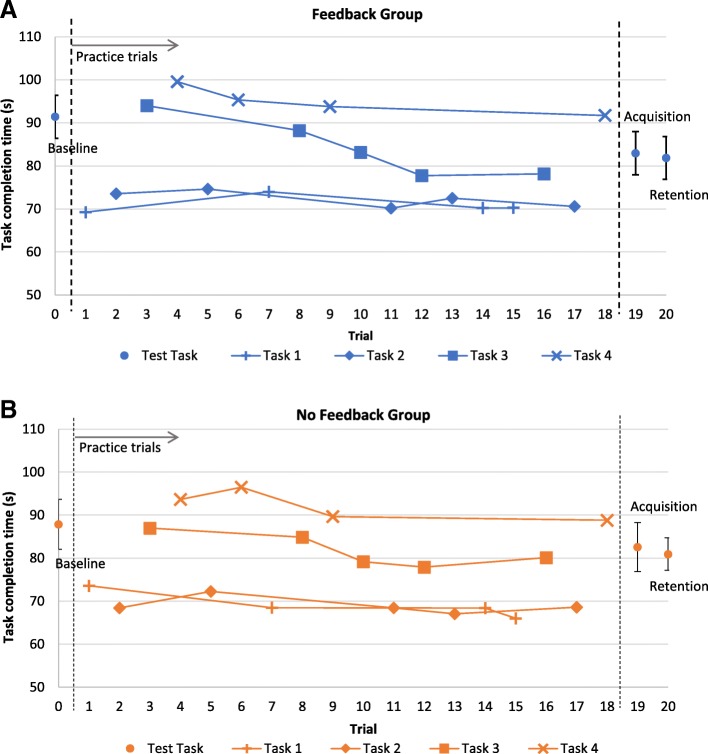
Mean task completion time results across trials according to task version for the **a**) Feedback group and **b**) No feedback group. Error bars representing confidence intervals are shown for the test task only. Confidence intervals for the other tasks were similar and are not shown for clarity. Task completion time generally decreased over the course of training

Figure 7 shows the mean task completion times obtained for the different trials (including the test tasks) for the feedback group (Fig. 7a) and the no feedback group (Fig. 7b). It can be seen that completion time generally decreased during training for each of the task’s difficulty levels.

**Fig. 8:**
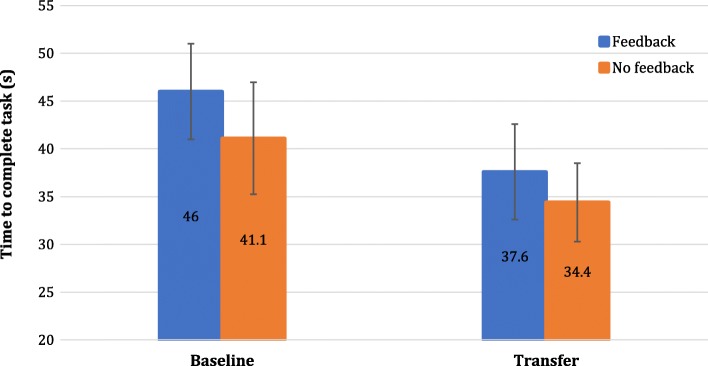
Number of collisions in the VE for feedback and no feedback group at baseline, acquisition and retention. Overall, participants had better performance from baseline to acquisition and the effect was maintained at retention. No significant group differences were found. Error bars represent confidence intervals

The results for the number of collisions in the VE test task are shown in Fig. 8. Participants made fewer collisions on the acquisition test 20 min after training and the effect was retained for the retention test. The mixed effects model was significant for time from baseline to acquisition (*p* = .001) and from baseline to retention (*p* = 0.01). No significant group differences were found.

### RE transfer test

**Fig. 9:**
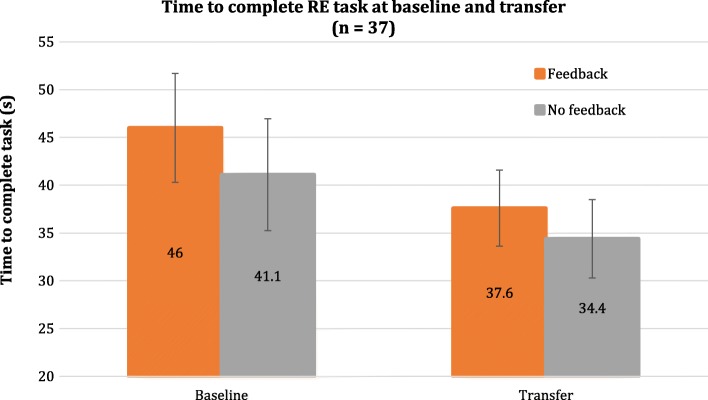
Time to complete the RE test task for all participants in the feedback and no feedback groups, at baseline and for the transfer test two days after training. Overall, participants had better performance from baseline to the transfer test. No significant group differences were found. Error bars represent confidence intervals

The results for the time to complete the RE test task are shown in Fig. 9. Three participants did not complete the real driving task because of technical problems with the PW. Similarly to the VE test task, the control group had better baseline performance than the feedback group. Overall, participants improved their real PW driving by 7.5 s two days after training. The mixed effects model found that this effect was significant (*p* < .001). However, the effect of feedback was not significant (*p* = .202).

Not enough collisions were made in the RE to see an effect.

### Speed-accuracy distribution differences

Contrary to our hypothesis, no significant group differences were found for time to complete the VE or RE test tasks and the number of collisions. However, an important factor to consider is that time and collisions influence each other, such that driving faster might cause more collisions and focusing on avoiding collisions may lead to slower driving. The instructions given to participants did not emphasize one strategy over the other; both speed and collisions were emphasized equally. Participants were told to drive as fast as possible while avoiding collisions. It is thus possible that participants used different strategies: some may have focused on their speed, others may have prioritized careful driving to avoid collisions, and the rest may have tried to focus on speed and collisions equally. Such speed-accuracy tradeoffs are common in perceptual-motor tasks and can make independent comparisons on speed or accuracy difficult. Therefore, while no group differences were found for time to complete task and number of collisions independently, differences may exist when taking into account their interaction.

**Fig. 10:**
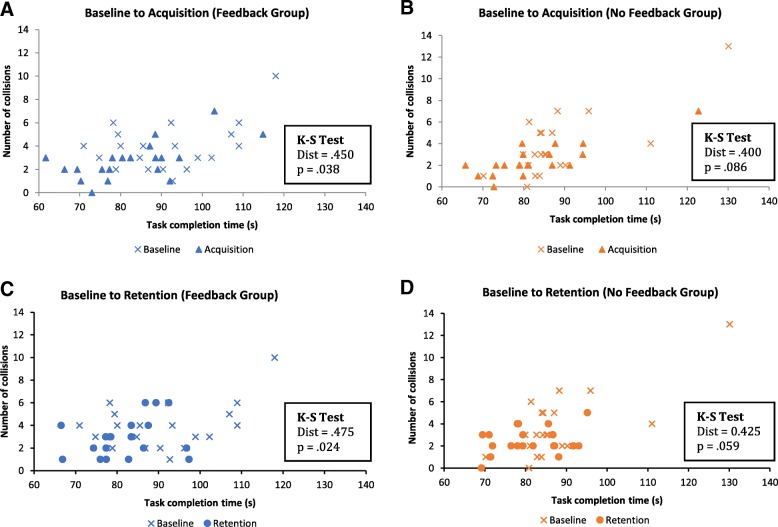
Speed-accuracy distributions for the VE test task. **a**. Comparison of the baseline and acquisition distributions for the feedback group. **b**. Comparison of the baseline and acquisition distributions for the no-feedback group. **c**. Comparison of the baseline and retention distributions for the feedback group. **d**. Comparison of the baseline and retention distributions for the no feedback group. For baseline to acquisition, the 2D KS test is significant for the feedback group but not for the no-feedback group, indicating that participants in the feedback group had better speed and accuracy after training

To account for this, a graph of collisions over time was plotted for the VE test task at baseline, acquisition, and retention where each participant represents a single point on the graph (Fig. 10).

A two-dimensional version of the Kolmogonov-Smirnov test (2D KS) [20] was used to analyze the differences between the distributions. The 2D KS is a non-parametric test that determines whether two samples have the same distribution by looking at the difference in the number of occurrences of each x-y pair in each quadrant of the graph.

The speed-accuracy distributions for the two groups overlap at baseline, acquisition and retention (not shown), and are not significantly different from each other.

We expect this based on the results from the mixed effects model: both groups perform similarly at baseline, acquisition and retention for both task completion time and number of collisions.

When looking at the two groups individually, differences are found. The 2D KS test is significant for the feedback group from baseline to acquisition (Fig. 10a), indicating that the speed-accuracy distributions before and after training were different. Participants in the feedback group drove faster and/or made fewer collisions after training. Conversely, although the control group did improve in speed-accuracy, before and after training, the differences did not reach significance (Fig. 10b).

This effect was somewhat maintained for the retention test. The feedback group’s speed-accuracy distributions from baseline to retention were significantly different. As for the control group, this difference in distributions was similar to the one obtained for the feedback group and almost reached significance (2D KS test; *p* = .059). This suggests that feedback does appear to play a role improving speed and accuracy during training but that its effect may not be strong enough to be maintained two days after training and can only be seen immediately after training.

**Fig. 11:**
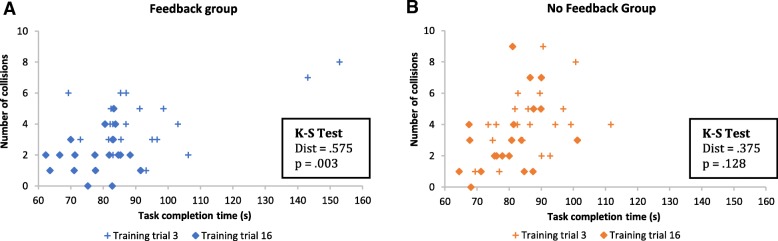
Speed-accuracy distributions for the VE training task number 3. **a**. Comparison of training trials 3 and 16 distributions for the feedback group. **b**. Comparison of training trials 3 and 16 distributions for the no-feedback group. The 2D KS test is significant for the feedback group but not for the no-feedback group, indicating that participants in the feedback group had better speed and accuracy at the end of training in comparison to the beginning of training

To further investigate this idea, we looked at the speed-accuracy distributions for one of the training tasks, task number 3 (Fig. 11). Task number 3 was a difficult task that appeared five times over the 18 training trials. For this analysis, we compared the first time task 3 was performed by the participants (trial 3) to the last trial it was performed (trial 16). Similar to what was found for the test task, looking at each group individually shows a significant difference in the speed-accuracy distributions from trial 3 to trial 16 for the feedback group (*p* = .003). That difference is not significant for the no feedback group (*p* = .128).

The low *p*-value obtained for the feedback group for this training task suggests that feedback does improve speed and accuracy and is most effective during training.

Not enough collisions were made with the real PW driving task to investigate their speed-accuracy tradeoff distribution.

## Discussion

Overall, simulator training improved participants’ PW driving performance in terms of time and number of collisions from baseline to acquisition in VE, and the effects seen at acquisition were retained two days later. Simulator training also improved participants’ real PW performance, from baseline to the transfer test two days after training for task completion time. Therefore, similar to what has been found in other studies [16], training in a PW simulator is effective in improving real PW driving skills.

No significant group differences were found for task completion time and number of collisions for the acquisition, retention and transfer tests. However, significant differences were found in the speed-accuracy tradeoff distributions for the feedback group from baseline to acquisition and during training with task number 3. The low *p*-value obtained for the feedback group for this training task suggests that feedback did improve speed and accuracy and was most effective during training. Its effect was maintained at the acquisition test 20 min after training but was not seen two days after training, where control of speed-accuracy improved to a similar degree in both the feedback and the no-feedback groups.

### Weak effect of augmented feedback: Not informative enough

Although simulator training was found to improve performance in both VE and RE, the effect of augmented feedback was not as strong as what was hypothesized based on the motor learning literature. One possible explanation for the lack of a strong effect of augmented feedback on PW driving performance is that the feedback strategy chosen for this experiment may not have been strong enough. No previous study has investigated the role of augmented feedback for PW driving. Most of the motor learning literature is based on experiments conducted with simple laboratory tasks (e.g. making a reaching movement with a robotic arm). Complex tasks have more degrees of freedom, are more difficult to master, are more cognitively demanding (planning, memory, attention) and are more ecological [21]. PW driving requires the acquisition of several skills, such basic right and left turns and more challenging skills such as maneuvering in constrained areas and negotiating fixed and moving obstacles [17]. PW driving may thus be qualified as a complex ecological task, compared to what has traditionally been studied in motor learning research. An increasing number of studies are applying concepts derived from simple tasks to more complex tasks and are finding contradictory results. For example, the guidance hypothesis, derived from experiments with simple tasks, was found not to be generalizable to more complex tasks. According to the guidance hypothesis, the purpose of augmented feedback is to guide learners to the correct response during the acquisition phase of learning. Providing too much of it can be detrimental to long-term learning because learners can become dependent on the feedback and fail to use their own error-detection mechanisms [9, 22]. In such cases, superior performance is seen during practice when feedback is present, but results in poorer performance on a retention test once feedback is removed [23, 24]. Therefore, it was suggested that decreasing the amount of augmented feedback during training results in better long-term learning.

Studies did not find that reducing augmented feedback improved long-term learning for complex tasks [21, 25]. In some cases, they found that complex skill learning was improved by more augmented feedback. Therefore, for complex tasks, providing terminal information feedback regarding their outcome may not be informative enough for the learner to modify the movement on a future trial [26, 27]. It is thus possible that the feedback given to participants regarding their time and number of collisions was not informative enough for them to make significant improvement on subsequent trials. Alternative feedback methods could include concurrent feedback during training with warning sounds when the PW is close to obstacles or feedback regarding joystick movement patterns.

### Weak effect of augmented feedback: Intrinsic feedback sufficient to improve performance

Since PW users will eventually use the simulator for training, we felt it was important for this study to simulate an authentic learning environment. Therefore, in comparison to some motor learning studies looking at the effect of feedback, participants were provided instructions regarding the learning goal and were not deprived of intrinsic feedback. Realistic sound effects were present in the VE, so that participants could detect when they made collisions. Participants could detect their errors even without augmented feedback and use this information on subsequent trials. This could explain in part why the differences between the groups were not as large as what was expected.

### Weak effect of feedback: Test task not sufficiently difficult

It is also possible that the task that was used for the tests in the VE was too easy, especially when considering the population that was tested. All participants were young adults with a university education. The test task might not have been of a high enough difficulty level to discern more subtle differences in performance. A better task to use as the test for this population might have been task version three, a difficult task for which it was possible to see consistent improvement during training. The test task that was used for the study was perhaps better suited for people with mobility impairments who require the use of a PW. These people tend to be older and often have other disabilities [28] which can make PW skill learning more difficult than for healthy controls.

### Weak effect of augmented feedback: High skill level of participants

One issue with the experiment is that although both groups were evenly distributed in terms of gender, age and education, the control group had better baseline performance than the feedback group. Because the control group’s performance was already good at baseline, it is possible that feedback was not useful or needed for most people in this group and therefore the lack of feedback had no impact on their performance. Interestingly, all participants except one in the feedback group improved in their time to complete the task from baseline to retention for the VE test task, whereas five out of twenty participants in the no feedback group had worse performance on the VE retention test than at baseline. This suggests that there were some people in the control group for whom feedback might have been useful.

### Motivation

Motivation is an important aspect of skill acquisition. The augmented feedback group could compare their time and number of collisions after each trial and use the information to motivate themselves to do better on future trials while the control group did not have access to this information. Because no significant group differences were found, motivation did not appear to play a large role.

### Limitations of study

One important limitation of the study is the non-immersive nature of the virtual environment. The goal of training in a VE is to replicate the experience of real PW driving, and a desktop monitor VE may not be as realistic as head-mounted displays (HMDs) which create a more immersive experience. In a recent study [29], PW training with a HMD was found to improve real PW driving performance to a greater extent than training using a non-immersive desktop VE. On the other hand, the use of an HMD may increase discomfort (nausea, vertigo, headaches) [30]; they could also be less appropriate for people with disabilities, who may have difficulties in donning such devices. Other simulators described in the literature include additional elements of realism and immersion, such as a moving platform to simulate inclinations [31], multiple screens [32], etc. Our approach with the miWe simulator has been to favor affordability and portability, to allow greater use in a clinical or home setting. We focus on ecological activity design and in providing useful feedback to the participants. Future research is needed to understand the impact of realism, immersion and feedback on wheelchair skill learning.

Another limitation of the study is the limited set of metrics used to measure PW driving performance. More detailed measures of accuracy such as PW path error, path smoothness and measures of joystick control might have allowed us to better discern the effect of feedback on PW driving performance and would be interesting to measure in a future study.

An additional limitation to the study is the small sample size. Feedback is maybe more useful for some people than others (e.g. baseline skill level). However, there were not enough participants to investigate patterns in the data.

## Conclusion

Training in a simulator results in retention and transfer of PW skills. There was a small effect of feedback seen when looking at the differences in the speed-accuracy distributions before and after training. The lack of a strong effect of feedback may be due to the population tested, the task difficulty and the strength of the feedback chosen for the experiment. Future studies should include testing with a different population (PW users, people with disabilities), adapting the task difficulty to the population and providing more informative feedback.

## Abbreviations

2D KS: Two-dimensional version of the Kolmogonov-Smirnov test
miWE: McGill Wheelchair Simulator
PW: Powered wheelchair
RE: Real environment
VE: Virtual environment
VR: Virtual reality

## References

[1] Smith EM, Giesbrecht EM, Mortenson WB, Miller WC (2016). Prevalence of wheelchair and scooter use among community-dwelling Canadians. Phys Ther.

[2] Edwards K, McCluskey A (2010). A survey of adult power wheelchair and scooter users. Disabil Rehabil Assist Technol.

[3] Torkia C, Reid D, Korner-Bitensky N, Kairy D, Rushton PW, Demers L, Archambault PS (2015). Power wheelchair driving challenges in the community: a users’ perspective. Disabil Rehabil Assist Technol.

[4] Pithon T, Weiss T, Richir S, Klinger E (2009). Wheelchair simulators: a review. Technol Disabil.

[5] Archambault PS, Tremblay S, Cachecho S, Routhier F, Boissy P (2012). Driving performance in a power wheelchair simulator. Disabil Rehabil Assist Technol.

[6] Bilodeau EA, Bilodeau IM (1961). Motor-skills learning. Annu Rev Psychol.

[7] Ammons RB (1956). Effects of knowledge of performance: a survey and tentative theoretical formulation. J Gen Psychol.

[8] Magill RA (1994). The influence of augmented feedback on skill learning depends on characteristics of the skill and the learner. Quest.

[9] Salmoni AW, Schmidt RA, Walter CB (1984). Knowledge of results and motor learning: a review and critical reappraisal. Psychol Bull.

[10] Schmidt RA (1982). Motor control and learning : a behavioral emphasis.

[11] Schmidt RA, Lee TD (2014). Motor learning and performance : from principles to application.

[12] Schmidt RA, Bjork RA (1992). New conceptualizations of practice: common principles in three paradigms suggest new concepts for training. Psychol Sci.

[13] Adelola IA, Cox SL, Rahman A (2009). Virtual environments for powered wheelchair learner drivers: case studies. Technol Disabil.

[14] Harrison A, Derwent G, Enticknap A, Rose FD, Attree EA (2002). The role of virtual reality technology in the assessment and training of inexperienced powered wheelchair users. Disabil Rehabil.

[15] Linden MA, Whyatt C, Craig C, Kerr C (2013). Efficacy of a powered wheelchair simulator for school aged children: a randomized controlled trial. Rehabil Psychol.

[16] Lam J-F, Gosselin L, Rushton PW. Use of virtual technology as an intervention for wheelchair skills training: a systematic review. Arch Phys Med Rehabil. 2018;99:2313–41. 10.1016/j.apmr.2018.02.007 29530515

[17] Holliday PJ, Mihailidis A, Rolfson R, Fernie G (2005). Understanding and measuring powered wheelchair mobility and manoeuvrability. Part I. Reach in confined spaces. Disabil Rehabil.

[18] Guadagnoli MA, Lee TD (2004). Challenge point: a framework for conceptualizing the effects of various practice conditions in motor learning. J Mot Behav.

[19] Bagiella E, Sloan RP, Heitjan DF (2000). Mixed-effects models in psychophysiology. Psychophysiology.

[20] Press WH, Teukolsky SA (1988). Kolmogorov-Smirnov Test for Two‐Dimensional Data: How to tell whether a set of (x, y) data paints are consistent with a particular probability distribution, or with another data set. Comput Physics.

[21] Wulf G, Shea CH (2002). Principles derived from the study of simple skills do not generalize to complex skill learning. Psychon Bull Rev.

[22] Schmidt RA. Frequent Augmented Feedback Can Degrade Learning: Evidence and Interpretations. In: Requin J., Stelmach G.E. (eds) Tutorials in Motor Neuroscience. NATO ASI Series (Series D: Behavioural and Social Sciences). Dordrecht: Springer. 1991;62:59-75.

[23] Winstein CJ (1991). Knowledge of results and motor learning--implications for physical therapy. Phys Ther.

[24] Winstein CJ, Schmidt RA (1990). Reduced frequency of knowledge of results enhances motor skill learning. J Exp Psychol Learn Mem Cogn.

[25] Wulf G, Shea CH, Matschiner S (1998). Frequent feedback enhances complex motor skill learning. J Mot Behav.

[26] Lai Q, Shea CH (1999). The role of reduced frequency of knowledge of results during constant practice. Res Q Exerc Sport.

[27] Wulf G, Shea CH (2004). Understanding the role of augmented feedback. Skill acquisition in sport: Research, theory and practice.

[28] Arim R. A profile of persons with disabilities among Canadians aged 15 years or older, 2012. Canadian Survey on Disability, 2012. Volume Catalogue no. 89-654-X: Statistics Canada; 2015.

[29] John NW, Pop SR, Day TW, Ritsos PD, Headleand CJ (2018). The implementation and validation of a virtual environment for training powered wheelchair manoeuvres. IEEE Trans Vis Comput Graph.

[30] Hernandez-Ossa KA, Longo B, Montenegro-Couto E, Romero-Laiseca MA, Frizera-Neto A, Bastos-Filho T (2017). Development and pilot test of a virtual reality system for electric powered wheelchair simulation.

[31] Niniss H, Nadif A. Simulation of the behaviour of a powered wheelchair using virtual reality The Third International Conference on Disability, Virtual Reality and Associated Technologies. Alghero, Sardinia, Italy; 2000. http://www.icdvrat.org/2000/papers/2000_02.pdf.

[32] Devigne L, Babel M, Nouviale F, Narayanan VK, Pasteau F, Gallien P (2017). Design of an immersive simulator for assisted power wheelchair driving.

